# Microelement strontium and human health: comprehensive analysis of the role in inflammation and non-communicable diseases (NCDs)

**DOI:** 10.3389/fchem.2024.1367395

**Published:** 2024-03-28

**Authors:** Xin Ru, Lida Yang, Guohui Shen, Kunzhen Wang, Zihan Xu, Wenbo Bian, Wenqi Zhu, Yanzhi Guo

**Affiliations:** ^1^ Institute of Food and Nutrition Development, Ministry of Agriculture and Rural Affairs, Beijing, China; ^2^ College of Nursing, Mudanjiang Medical University, Mudanjiang, China; ^3^ Zibo Agricultural Science Research Institute, Shandong, China; ^4^ Digital Agriculture and Rural Research Institute of CAAS (Zibo), Shandong, China; ^5^ Agricultural Information Institute, Chinese Academy of Agricultural Sciences, Beijing, China; ^6^ Chinese Academy of Agricultural Sciences, Beijing, China

**Keywords:** microelement, strontium, non-communicable diseases, inflammation, human health

## Abstract

Strontium (Sr), a trace element with a long history and a significant presence in the Earth’s crust, plays a critical yet often overlooked role in various biological processes affecting human health. This comprehensive review explores the multifaceted implications of Sr, especially in the context of non-communicable diseases (NCDs) such as cardiovascular diseases, osteoporosis, hypertension, and diabetes mellitus. Sr is predominantly acquired through diet and water and has shown promise as a clinical marker for calcium absorption studies. It contributes to the mitigation of several NCDs by inhibiting oxidative stress, showcasing antioxidant properties, and suppressing inflammatory cytokines. The review delves deep into the mechanisms through which Sr interacts with human physiology, emphasizing its uptake, metabolism, and potential to prevent chronic conditions. Despite its apparent benefits in managing bone fractures, hypertension, and diabetes, current research on Sr’s role in human health is not exhaustive. The review underscores the need for more comprehensive studies to solidify Sr’s beneficial associations and address the gaps in understanding Sr intake and its optimal levels for human health.

## 1 Introduction

Strontium (Sr), a trace element with a storied history and widespread presence on Earth, plays a significant yet often understated role in various geological and human biological processes (Piette et al., 1994). Predominantly located in the Earth’s crust, Sr has been a subject of scientific curiosity since its discovery in 1790 (Gad, 2014). Over time, its presence has been noted in various natural resources, including soil, minerals, and seawater as one of the essential trace elements in the human body at low concentrations of about 10.57–12.23 mg/L (Varo et al., 1982; Piette et al., 1994; Burger and Lichtscheidl, 2019). Sr is mostly deposited in human bones, 99% of strontium is precipitated in the femur, lumbar spine, and iliac bone, while the remaining 0.7% is found in extracellular fluid (Cai et al., 2021). Derived from a broad array of sources, Sr is mainly consumed through food and water (Marie et al., 2001). In clinical trials of intestinal, strontium offers the potential as a marker to test parameters for measuring calcium absorption (Sips et al., 1995; Sips et al., 1996). Studies have shown that overall discrimination is quite high for animals on a high-strontium diet (HARRISON et al., 2009). Empirical studies have established that strontium not only mitigates oxidative stress but also exhibits pronounced antioxidant capabilities. Additionally, it plays a crucial role in suppressing the release of pro-inflammatory cytokines, thereby exerting significant anti-inflammatory effects. (Zhu et al., 2016).

Sr’s relevance extends to the realm of non-communicable diseases (NCDs), which are the leading cause of morbidity worldwide and account for seven out of every ten global deaths (Bennett et al., 2018). The role of Sr in mitigating NCDs such as cardiovascular disease, osteoporosis (Dahl et al., 2001), hypertension (Barneo-Caragol et al., 2018a), diabetes (Hellman et al., 1997; Maehira et al., 2011) and a large number of population samples have demonstrated that strontium plays a, contributing role in the amelioration of all of the above diseases (Marie, 1996; Chen et al., 2020; Baheiraei et al., 2021; Xing et al., 2021; Wang et al., 2022). According to estimates from The *Lancet*, NCD-related deaths increased from 80.0% in 2002 to 88.5% in 2019, with major contributors being cardiovascular diseases (47.1%), cancer (24.1%), and diabetes (2.5%) being the major contributors. The burden of disease in China has been shifting from infectious to non-communicable diseases (Bennett et al., 2018). The prevalence of hypertension increased from 25.7% in 2007, to 31.5% in 2017 and the prevalence of diabetes is expected to escalate significantly, reaching a staggering 257 million cases by the year 2050 (Ong et al., 2023; Yip et al., 2023). The escalating prevalence of non-communicable diseases (NCDs) profoundly influences population health, national economic stability, and the efficacy of healthcare systems. Concurrently, the expansive research into Sr has revealed its substantial potential in alleviating the impact of these chronic conditions on global health. Historically, the study of Sr has traversed diverse fields, from geochemistry to biomedicine, reflecting its versatile nature and importance.

**FIGURE 1:**
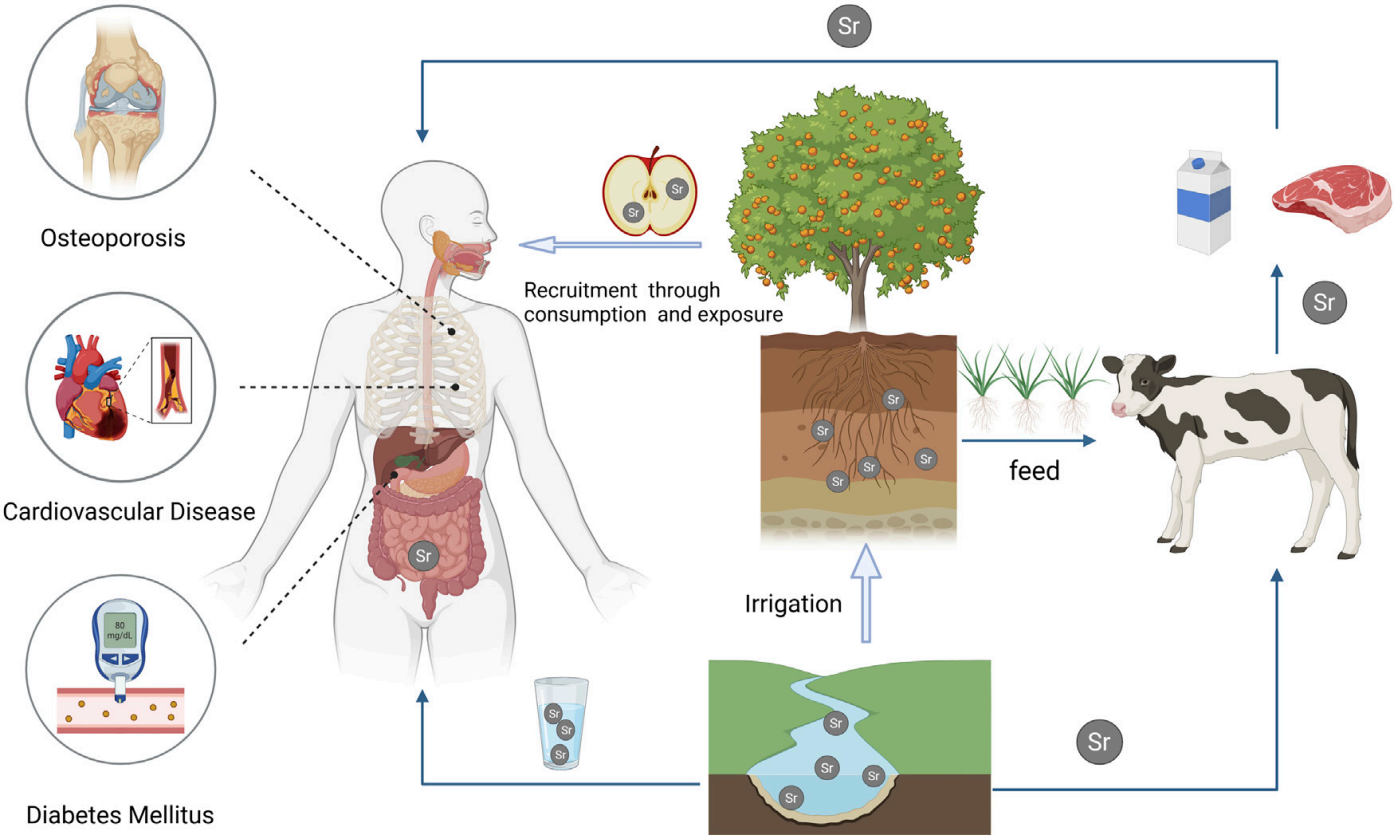
This illustration depicts the assimilation of strontium from the environment into agricultural products via natural water sources and soil. Humans acquire essential strontium through the consumption of strontium-enriched animal and plant-based foods or by drinking natural mineral water containing strontium. Extensive research indicates the effectiveness of strontium in inhibiting the production of inflammatory factors and improving the management of non-communicable chronic diseases.

The world is currently grappling with an unprecedented crisis in micronutrient deficiencies, a condition often referred to as ‘hidden hunger’ which can coexist within the same population impacting over two billion people worldwide and acknowledging the global challenge of hidden hunger and the crucial role of micronutrients in human health (Black et al., 2013; Grebmer et al., 2014; Lowe, 2021). Acknowledging the global challenge of hidden hunger and the crucial role of micronutrients in human health. The onset of most non-communicable diseases (NCDs), including autoimmune, metabolic, cardiovascular diseases, and cancer, has been correlated with metals, metalloids, and the excess or deficiency of essential oligo-elements in the body. These elements are ubiquitous and the body is constantly exposed to them via their presence in soil, water, air, and food (López-Abente et al., 2018; Ali et al., 2019). This review aims to offer a comprehensive overview of the biological functions of Sr, pathways of intake, metabolism, and implications for various diseases, emphasizing its significance and potential in addressing pressing health concerns. The decision to focus on Sr stems from its emerging significance in NCDs of its mechanisms and effects. Through an exploration of the diverse roles and potential therapeutic advantages of Sr, this review aims to offer valuable insights into the potential of Sr for addressing nutrient deficiencies and NCDs, while making a meaningful contribution to the development of public health strategies and interventions (Figure 1).

## 2 Presence and distribution of strontium in environment

**FIGURE 2:**
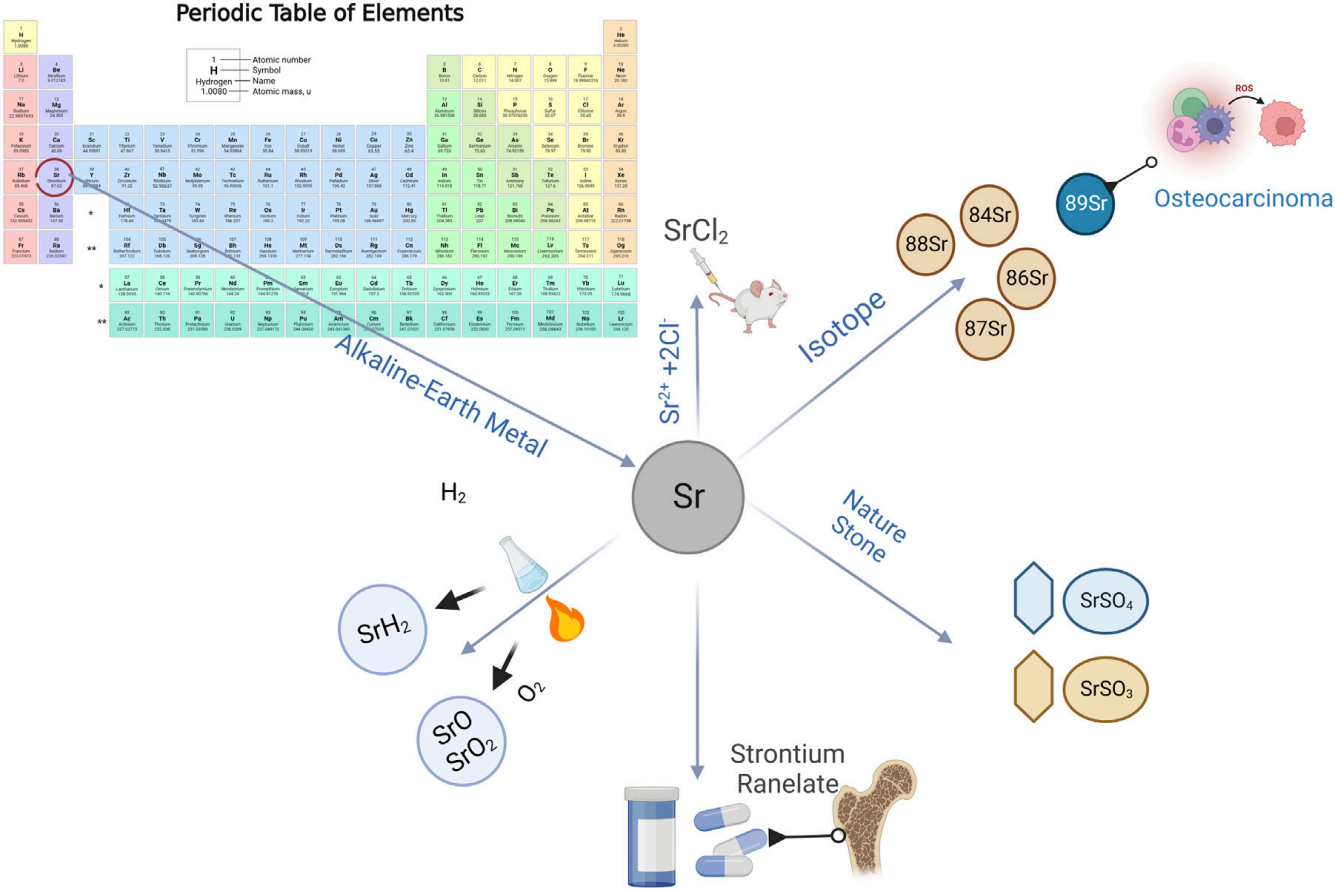
Multiple forms of strontium in the natural environment.

Sr is an alkali-earth metal element located in the fifth cycle, group IIA, between calcium and barium. Its physicochemical properties bear resemblance to those of calcium and barium, though it also possesses unique characteristics distinct from these elements. Sr metal is readily oxidized to form Sr oxide, which exhibits a pale-yellow color (Murray, 1993). The primary sources of Sr are Sr sulfate (SrSO_4_) and Sr carbonate (SrCO_3_). The natural Sr element is a mixture of four stable isotopes (Shand et al., 2009): ^84^Sr (0.56%), ^86^Sr (9.86%), ^87^Sr (7.02%) and ^88^Sr (82.56%). It is made up of the radioactive alkali metal rubidium (^87^Rb) decay, which has a half-life of 4.88 × 10^10^ year. The source of ^87^Sr comes from two pathways. One is associated during the original nucleosynthesis with ^84^Sr, ^86^Sr and ^88^Sr formed together, and the other one is given by ^87^Sr. Formation by the radioactive decay of Rb. Usually reported in geological surveys ^87^Sr/^86^Sr, to indicate the source of Sr in environmental samples, is used to identify sediment areas in marine and river environments (Bu et al., 2016; Ryan et al., 2018). The isotopes of Sr are radioactive, such as ^90^Sr, which was used as a tracer compound (Pathak, 2017; Pathak and Sharma, 2018). ^90^Sr first decays to yttrium (^90^Y) and is eventually converted to zirconium after completing its life cycle (^90^Zr) (BU et al., 2016). A medium content of Sr from 0.02%–0.03% in the earth’s crust, which the main source of Sr element way is water. The average concentration of strontium in freshwater globally is 0.5–1.5 mg/L. (Peter, 2000; United States, 2004). (Figure 2)

Sr is ubiquitous in the environment and spread throughout almost all rocks and soils. Its presence is greater than the presence of carbon or chlorine and slightly lower than sulfur or fluorine. The average concentration of stable Sr in the soil is about 240 ppm, but Sr in agricultural soils may exceed 600 ppm, such as in soil treated with phosphate fertilizer or limestone (Bohn et al., 1979). The release of Sr from the earth’s crust into the atmosphere is the result of natural processes, such as the entrainment of dust particles and the resuspension of the soil, which will lead to the release of Sr. Furthermore, many human activities will cause the release of Sr into the atmosphere, such as the grinding and processing of Sr compounds, burning coal, the application of phosphate fertilizers on land, and the use of pyrotechnic devices (Feng et al., 2016; Pathak et al., 2017). Translation with lower Sr content in natural water sources, such as rivers and wells, but they can vary greatly near dolomite-rich limestone deposits. Research was conducted on public drinking water samples collected from 314 cities across the country to assess the concentration and spatial distribution of strontium (Sr) in public drinking water. The Sr concentration ranged from 0.005 to 3.11 mg/L, with an average value of 0.360 mg/L. Significant regional variations were observed, with higher Sr concentrations generally found in the northern regions and lower concentrations in the southern areas (Peng et al., 2021).

Sr was not significant for the growth or reproduction of most plants while it is easily absorbed from the soil by the plants. Plant uptake of Sr occurs most in clay and sandy soils with low organic content, but the concentration of nutrient mineral elements (such as calcium) in the soil reduces the absorption of Sr by aboveground plants. Compared with root absorption, the leaves absorb very little Sr while the content of Sr in plants is very small (1 ppm–169 ppm, average 36 ppm dry weight) (Russell and Squire, 1958). Once absorbed in the plant, Sr is distributed to other parts of the plant, such as leaves or fruit. The distribution of Sr in plants is mainly in the leaves and fruits of translation. Therefore, Sr is a natural component of food (Qi et al., 2015). The extended survey showed that most foods had lower Sr, such as meat, poultry, potatoes, fruits, milk, and dairy products (fresh weight below 1 ppm), while cereals, root vegetables, and seafood had higher Sr (e.g., 3.7 ppm for bread, 3.6 ppm for fish, 1.6 ppm for green vegetables, and 1.3 ppm for cereals). The highest level can be found in the nuts (8.67 ppm) (Nabrzyski and Gajewska, 2002; Qi et al., 2015).

## 3 Strontium dynamics in human physiology: Absorption, distribution, and excretion

### 3.1 Absorption dynamics

Sr’s physiological journey begins in the jejunum, where it is primarily absorbed. This absorption is facilitated by calcitriol and Ca ions through the epithelial Ca channel 2 (ECaC2) or TRPV6 transporter (Barneo-Caragol et al., 2018b). Sr absorption is influenced by various factors: its absorption decreases relative to increases in Sr dosage, the Ca concentration in food, and individual age. Conversely, Sr absorption can be facilitated by increased levels of vitamin D in the body and higher concentrations of lactose and carbohydrates in food. The absorption methods also exhibit some variations, with Sr absorbed from aqueous solutions being more prevalent (approximately twice as much) compared to Sr absorbed from solid food sources. High-Ca diets and the intake of alginates have been demonstrated to inhibit Sr absorption. Alginates, a type of carbohydrate extracted from seaweed, have been found to significantly reduce Sr absorption in both humans and animals, without adversely affecting Ca absorption (Carr et al., 1968).

The main way to ingest Sr is through food and water. Sr is present in media as a salt or an ionized divalent cation. The Sr ion (dissociated) is toxicokinetic ally important because it is easily absorbed into systemic circulation when inhaled with particulates or ingested with water or foods (Melnyk et al., 2019). Drinking water and the urine levels of Sr have a significant negative correlation with hypertensive heart disease. There is a significant positive correlation between the sodium/Sr ratio in drinking water and the central nervous system vascular injury, arteriosclerosis, degenerative heart disease, and hypertensive heart disease while a significant negative correlation between urinary sodium/Sr ratio and systemic arteriosclerosis (Peng et al., 2021). The mechanism of action may be the competitive absorption of Sr with sodium in the intestine, thereby reducing sodium absorption and increasing sodium excretion in the body. Sr can reduce excessive sodium in the human body which has caused high blood pressure and cardiovascular disease, and inhibit the body’s absorption of sodium. In addition, it was found that the average concentration of Sr in drinking water in the longevity township was significantly higher than that of drinking water in the non-longevity township. There is an obvious regional longevity phenomenon in China, most of which are located in south China (Liu et al., 2018). Du et al. (2017), found that the concentration of Sr in drinking water in longevity areas of South China is between non-longevity areas of Southern China and North China. It can be inferred that the appropriate concentration of Sr in drinking water can play a more effective role in prolonging life.

The primary route of strontium into the body is through the gastrointestinal tract. The absorption of strontium in the gastrointestinal tract is age-dependent, ranging from approximately 90% in infants to around 10% in the elderly (Underwood, 1977). The absorption within a specific age group exhibits significant individual variations, linked to the functional state of the gastrointestinal tract (Likhtarev et al., 1975). Various mechanisms for strontium transport through the intestinal wall have been proposed. While some authors suggest passive diffusion (paracellular transport) as the exclusive absorption mechanism, others, including Papworth et al., (Corbett et al., 2014), propose two routes: carrier-mediated and diffusion-mediated. Generally, strontium is absorbed to a lesser extent than calcium, with a strontium/calcium absorption ratio of approximately at the end of the lactation period (Dumont et al., 1960; Karbach and Rummel, 1987; Milsom et al., 1987). These findings underscore the intricate dynamics of strontium absorption, influenced by age, individual variability, and various physiological conditions. Mechanistic insights into strontium transport, including both passive diffusion and proposed carrier-mediated routes, contribute to our understanding of strontium’s bioavailability (Patrick, 1967). Factors such as dietary composition, fasting, and physiological states like pregnancy and lactation play crucial roles, emphasizing the multifaceted nature of strontium absorption (Moore and Elder, 1965; Kostial et al., 1967). The research documented in this study was centered on juvenile rats, methodically evaluating two pivotal factors that are known to influence the differential absorption and retention of calcium and strontium. These factors are particularly the dietary levels of calcium and phosphorus, which are understood to play a significant role in modulating these processes (Author Anyonumus, 1969; Baheiraei et al., 2021). Additionally, alternative absorption routes via the lung and skin further enrich our comprehension of strontium’s complex uptake mechanisms (Sharma, 2020). The Sr content in human dietary intake, as well as the subsequent daily consumption, exhibits variability based on geographic locales and diverse food types (LV et al., 2011; LIU et al., 2018). The local environmental determinants, significantly influencing the Sr levels in potable water, also contribute considerably to Sr intake (Curzon et al., 1978; Fraga, 2005; Li et al., 2016). Insights derived from dietary surveys, food composition databases, and balance studies suggest that the daily Sr intake likely ranges from 1.4 to 5.7 mg. A comprehensive dietary investigation undertaken in the United Kingdom in 1994 estimated the dietary exposure to Sr at approximately 2–4 mg per day. Globally, it has been reported that the daily Sr intake averages around 1.5 mg (Pors Nielsen, 2004).

### 3.2 Distribution in the body

Once absorbed, about 99% of Sr is deposited in bone, connective tissue, and teeth, mirroring calcium’s distribution (Cai et al., 2021). Sr can substitute for calcium in the bone mineral matrix which is deposited in bone initially distributed on the bone surface of cortical or trabecular bone and exchanging relatively quickly with calcium (Ca) in plasma, or bone mass. Besides the bones, Sr is relatively abundant in the blood. In serum or plasma, Sr forms divalent cations combined with varying degrees of protein. Sr binds to proteins to the same extent as Ca in serum or plasma, and the binding rate may be between 20%–40% (Piette et al., 1994). The normal serum Sr content in adults with intact renal function ranges from 10 μg/L to 217 μg/L (Cabrera et al., 1999). It has been suggested that the positive effect of Sr on bone and tooth structure is mainly attributed to the interaction between Sr^2+^ and Ca^2+^ surrounding anions in the bone mineral structure (Marie et al., 2001). It has appeared that Sr displaces Ca to locally form Sr salts in bone structures, however increasing bone mineral density or protecting enamel from the dissolution in contact with the erosive environment during intake of food and beverages (Zhao et al., 2020). The way to increase Ca ion activity is measured electrochemically by adding Sr chloride to the aqueous suspension of the metastable Ca citrate tetrahydrate (Liu and Skibsted, 2022), and it has also been found to increase bone mineral density with Sr citrate intake (Siccardi et al., 2010). In North America, Sr citrate is approved as a food supplement and is sold in health food stores (Moise et al., 2012). A recent study reported that Sr citrate also prevented loss of enamel hardness under acidic conditions due to lower solubility and reduced solubility of the Ca/Sr mixed minerals (Zhao et al., 2020).

Sr is present in all organisms due to its relatively high concentration (about 400 ppm). The distribution and metabolism of Sr in the human body are similar to Ca. Both of them have “bone-seeking property,” the Sr in bone and blood constantly exchange with a dynamic balance (Panzavolta et al., 2018). The common daily Sr balance has been established as follows: intake with food/fluid—1.93 mg, secretion with urine—0.34 mg, excretion with feces—1.5 mg, secretion via sweat glands—0.02 mg and loss with hair—0.2 × 10^−3^ mg (Pors Nielsen, 2004; Filippini et al., 2020). Significant endogenous levels of strontium are present in human serum samples ranging from 19 to 96 ng/mL with a mean of 34.6 ± 15.2 ng/mL (SD) (Somarouthu et al., 2015).

### 3.3 Excretion and metabolic dynamics

The human body’s excretion pathways for different alkaline earth metals are diverse. Barium and radium are primarily excreted through feces, while Sr and Ca are mainly excreted through the kidneys, with minor amounts expelled via breast milk and feces. The renal tubular reabsorption rate for Sr is lower than that for Ca, resulting in Sr typically being excreted at a higher rate than Ca (Carr et al., 1968). The human body handles Sr in a manner similar to Ca; it is absorbed from the intestines, concentrates in the skeleton, and is primarily excreted through urine. Additionally, Sr can also be expelled in feces, breast milk, and sweat. Sr excreted in the feces comes from either unabsorbed orally ingested Sr or Sr actively excreted by the intestines, the latter being directly related to blood Sr concentrations. The Sr/Ca ratio is higher in urine than in the glomerular filtrate, indicating that these two elements are differentiated during the renal tubular reabsorption process in the kidneys. The daily urinary excretion in adults is estimated to be between 0.1 mg and 0.4 mg, with a total clearance rate varying between 9.4 and 11.7 mL/min. Sr can enter human breast milk and be transferred to newborns during breastfeeding. The average Sr concentration in the breast milk of healthy women is estimated to be approximately 74 mg/L.

## 4 Potential mechanisms of strontium in modulating inflammatory responses and Non-Communicable Chronic diseases

### 4.1 Interplay between inflammation and Non-Communicable Chronic diseases

Inflammation, the body’s natural defense against injury or infection, involves the activation of various immune cells, including macrophages, T cells, and B cells, accompanied by the production of cytokines (Hotamisligil, 2017). Contemporary research has illuminated a profound correlation between inflammatory responses and a spectrum of non-communicable chronic diseases (NCDs). This spectrum encompasses disorders ranging from arthritis, hypertension, metabolic syndrome, type 2 diabetes, cardiovascular diseases, obesity, cancer, and even pathologies related to sleep deprivation (Biondi-Zoccai et al., 2003; Medzhitov, 2008; Straub et al., 2010; Armstrong et al., 2013; López-Otín et al., 2013; Dregan et al., 2014; Besedovsky et al., 2019; Drummond et al., 2019; Schunk et al., 2021; Crea, 2022a; Crea, 2022b). A recurring theme in diseases that elicit inflammatory responses is the disruption of cellular and tissue homeostasis (Hotamisligil, 2006; Colaço and Moita, 2016). For instance, in hypertensive disorders, immune cells like cytotoxic T lymphocytes (CTLs), T helper cells (T_H_1), and B cells play pivotal roles in modulating cardiovascular dynamics (Drummond et al., 2019). CTLs, through the FASL-FAS system, induce apoptosis in target cells (Sanders and Wang, 2002), and their production of cytokines like interferon-gamma (IFNγ) and tumor necrosis factor (TNF) has been linked to hypertension and renal damage in mice models (Sriramula et al., 2008; Saleh et al., 2015). Obesity presents another illustrative example where lipid accumulation in adipocytes and hepatocytes triggers endoplasmic reticulum (ER) stress and inflammatory signaling, disrupting metabolic balance. The resulting unfolded protein response (UPR) intersects with inflammatory pathways such as NF-κB and oxidative stress networks (Hotamisligil, 2010). Metabolic syndrome, encompassing conditions such as insulin resistance and type 2 diabetes, is closely linked with chronic inflammation. This association is characterized by aberrant cytokine production, elevated levels of acute-phase reactants, and an increase in various other mediators. Additionally, there is an activation of a network of inflammatory signaling pathways, further illustrating the complex interplay between metabolic dysregulation and inflammation (Hotamisligil, 2005). Tumor necrosis factor-alpha (TNF-α), a key pro-inflammatory cytokine, is implicated in various signal transduction pathways. Its overexpression in the adipose tissue of obese mice is linked to obesity, diabetes, and chronic inflammation. Knockout mouse models lacking TNF-α function show improved insulin sensitivity and glucose homeostasis, highlighting TNF-α′s role in modulating insulin action in obesity (Hotamisligil et al., 1993; Uysal et al., 1997; Ventre et al., 1997). Furthermore, the widespread use of anti-TNF-α treatment in inflammatory diseases like rheumatoid arthritis has yielded definitive secondary outcomes. These outcomes support the role of TNF-α in systemic insulin sensitivity in humans, highlighting its significance in the pathophysiology of these conditions (Kiortsis et al., 2005; Gonzalez-Gay et al., 2006).

### 4.2 Strontium’s regulatory role in inflammatory responses and immune cell dynamics

Emerging evidence accentuates the critical role of metal elements in the structural integrity of inflammatory cells, catalytic processes, and signal transduction pathways (Hänsch and Mendel, 2009). Trace nutrients are deeply embedded in almost every aspect of human metabolism and cellular functionality (Wang et al., 2020). Historically, the physiological implications of these elements were initially characterized based on symptoms associated with their deficiency. These elements are indispensable as cofactors for enzymes, either as prosthetic groups or coenzymes, and are vital for the activation or mimicry of ion channels and secondary signaling pathways (Whitaker et al., 2021).

Strontium, as an essential trace element, exerts a dynamic regulatory influence on immune cell dynamics, with profound implications for T cell behavior, cytokine production, and the NF-κB signaling pathway. Strontium’s assimilation by T cells, mirroring calcium’s pathway, significantly alters intracellular calcium concentrations, thereby impacting T cell responses. This modification in calcium signaling is crucial for the differentiation of T cells into regulatory T cells (Treg), fostering the production of anti-inflammatory cytokines such as TGF-β and IL-10. This mechanism plays a key role in mitigating excessive immune responses in autoimmune and chronic inflammatory diseases, predominantly through the activation of transcription factors like NFAT, essential for T cell differentiation and function (Zhang et al., 2016; Yuan et al., 2017). Furthermore, strontium can modulate the expression levels of key pro-inflammatory cytokines, including IL-1, IL-6, and TNF-α, central to the orchestration of inflammatory responses. These cytokines typically activate downstream pathways such as NF-κB, thereby amplifying the production of inflammatory mediators by immune cells (Dolgikh et al., 2016). The impact of strontium on the NF-κB signaling pathway is significant; by reducing pro-inflammatory cytokine production, strontium may attenuate NF-κB activation, consequently diminishing the expression of inflammatory mediators and mitigating inflammation (Marx et al., 2022). Additionally, strontium’s role in regulating RANKL, a critical factor in bone metabolic balance that promotes osteoclast formation and activation, is of particular interest. Strontium may modulate RANKL expression produced by T and B cells, indirectly influenced by the reduction of pro-inflammatory cytokine levels, thus impacting bone inflammation and resorption processes (You et al., 2022).

Emerging research underscores strontium’s potential in treating various inflammatory diseases, notably through the reduction of pivotal inflammatory cytokines such as TNF-α and IL-6, key players in both inflammatory and autoimmune pathologies (Fromigué et al., 2009; Buache et al., 2012; Liu et al., 2021). The enhancement of anti-inflammatory cytokine production, like interleukin-10 (IL-10), by strontium further indicates its capacity to regulate immune balance and suppressing inflammatory responses. Strontium’s utility in treating osteoporosis also warrants attention. Combined administration of Sr-Ca effectively reduced TNF-α expression in a large animal model of osteoporosis (Wirsig et al., 2022). Buache et al. (2012), discovered that Sr-substituted biphasic calcium phosphate (Sr-BCP) can reduce the production of inflammatory cytokines, such as TNF-α and IL-6, while concurrently slowing osteoclast genesis. Complementarily, Fromigué et al., 2009, demonstrated that strontium ranelate effectively prevents osteoblast apoptosis induced by serum deprivation or pro-inflammatory cytokines IL-1β and TNF-α. Additionally, the suppression of chondrocyte apoptosis and mitigation of inflammatory responses by strontium salts has been corroborated in transcriptomic research studies (Liu et al., 2021). In experimental research, strontium salts have demonstrated significant anti-inflammatory effects. Topal et al (2014), the study noted that oral administration of strontium chloride hexahydrate in a murine model of ulcerative colitis significantly inhibited serum TNF-α levels, with therapeutic effects comparable to prednisone (Hahn, 1999; Li et al., 2016). A clinical randomized controlled trial revealed a significant negative correlation between serum TNF-α levels and serum Sr levels in perimenopausal women with normal BMI (Cybulska et al., 2021).

In a NAFLD mouse model, strontium mitigated hippocampal damage induced by a high-fat diet (HFD), potentially through mechanisms involving endoplasmic reticulum stress (ERS) or inflammation-related signaling pathways. It inhibited HFD-induced inflammation and improved hippocampal synaptic plasticity in NAFLD mice by blocking the TLR4/p38 MAPK/ERK, and NF-κB pathways (Tan et al., 2023). These findings indicate that Sr^2+^ can attenuate the expression of lipopolysaccharide-stimulated pro-inflammatory cytokines such as TNF-α, IL-1β, IL-6, and IL-8 (Wei et al., 2018). Sr has been employed in the treatment of a range of inflammatory conditions, including interstitial cystitis, temporomandibular joint osteoarthritis, allergic rhinitis, and neuroinflammation (Korgali et al., 2014). Topically applied inorganic strontium salts have shown remarkable effectiveness in inhibiting irritation and inflammation. This is particularly evident in their application for gingival inflammation and irritant dermatitis, positioning these salts as a novel class of selective inhibitors. In experimentally induced irritant contact dermatitis, strontium salts have consistently demonstrated anti-inflammatory effects, corroborating their therapeutic potential in inflammatory skin conditions. (Celerier et al., 1995; Hahn, 1999; Li et al., 2009; Portugal-Cohen et al., 2009; Altuntas et al., 2017; Yu et al., 2022; Zheng et al., 2022). Further extending its dermatological applications, strontium- and selenium-enriched thermal waters have proven effective in managing inflammatory skin diseases (Celerier et al., 1995). The therapeutic and cosmetic properties of Dead Sea mud and water, enriched with strontium, have been highly valued for their anti-inflammatory effects (Portugal-Cohen et al., 2009). Double-blind trials have substantiated the superior anti-inflammatory effects of strontium salts, highlighting their potential in skincare products to alleviate symptoms of irritant contact dermatitis (Fatemi et al., 2016). In a rat model of allergic rhinitis, nasal administration of strontium chloride effectively alleviated allergic symptoms (Altuntas et al., 2017).

**FIGURE 3:**
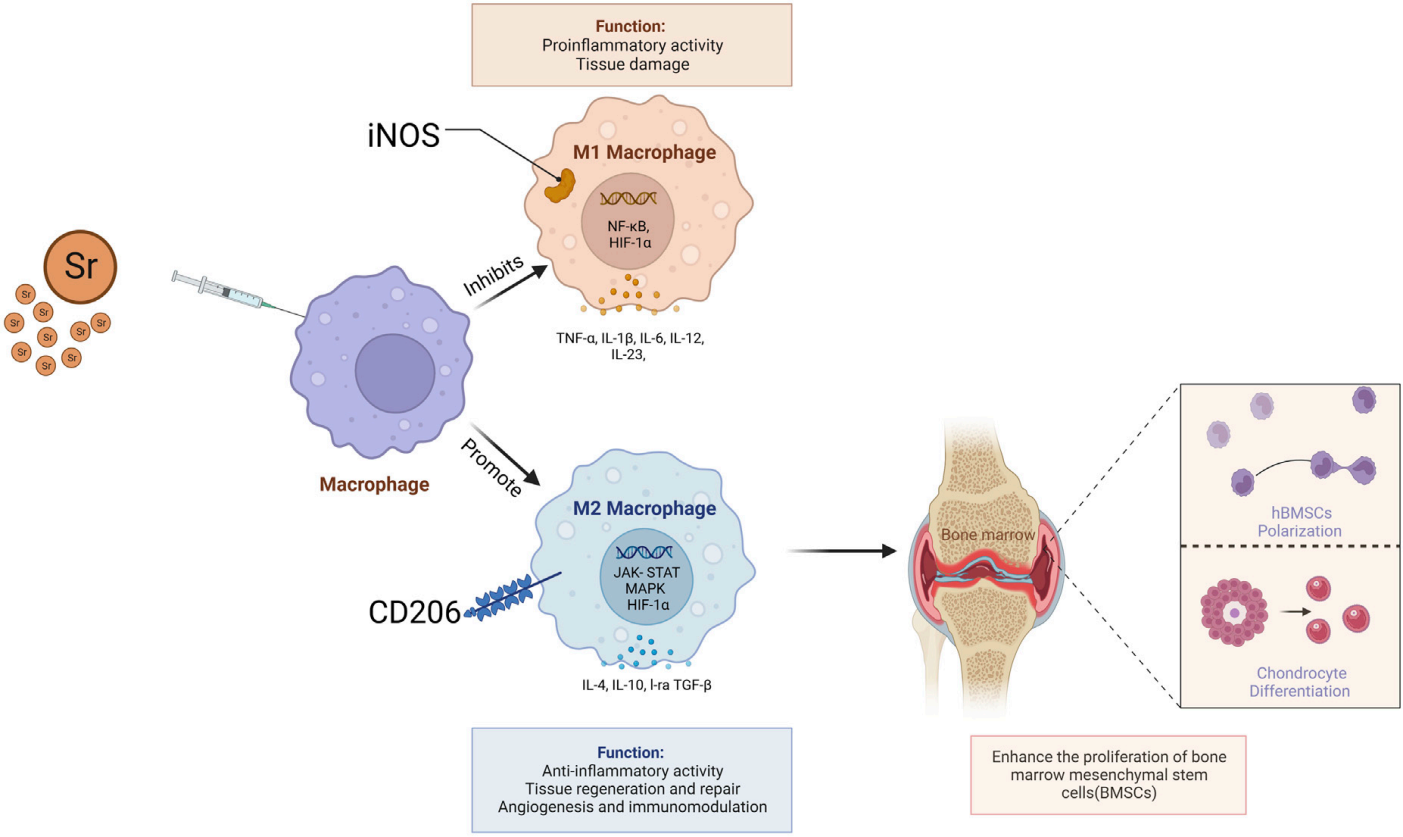
Strontium facilitates the regeneration of chondrocyte tissues and differentiation of human Bone Marrow Stromal Cells (hBMSCs) by promoting the secretion of anti-inflammatory cytokines such as IL-10 and arginase one from M2 macrophages.

Strontium has been studied for its potential to influence macrophage behavior, particularly its capacity to promote anti-inflammatory cytokine production. Macrophages, crucial in regulating inflammation, have two primary activation states: M1 and M2. M1 macrophages, known as classically activated inflammatory macrophages, create a pro-inflammatory environment by secreting cytokines like TNF-α, IL1β, and IL-6. Conversely, M2 macrophages, or activated inflammatory macrophages, secrete anti-inflammatory cytokines such as IL-10 and arginase 1, aiding in tissue regeneration and remodeling (Chazaud, 2014; Wynn and Vannella, 2016). (Figure 3) Research on Sr suggests it may encourage the M2-type macrophage response, thereby supporting an anti-inflammatory microenvironment. Cai et al. found that strontium enhances regenerative macrophage phenotypic expression and IL-10 production while inhibiting TNF-α expression, modulating the immune response (Choi and Park, 2018). SrBGM-treated RAW cells (macrophages) favored the M2 phenotype and exhibited enhanced expression of platelet-derived growth factor-BB (PDGF-BB). Furthermore, the conditioned medium from SrBGM-treated RAW cells significantly enhanced the angiogenic capacity of human umbilical vein endothelial cells (HUVECs) (Zhao et al., 2018).

### 4.3 Effects of strontium on osteoporosis

Investigations have pointed out that a low concentration of Sr was found in the femoral head, which is a common site for low-energy fractures (Burguera et al., 2002). Shorr and Carter, (1952), observed that the deposition of Ca in bones exceeded the total storage of Ca when a moderate dose of Sr lactate was given, in the absence of Sr. Subsequent research has demonstrated that Sr lactate can reduce bone pain in osteoporosis patients, with concurrent radiological signs of improvement. Reports indicate that Sr ranelate is capable of inhibiting bone resorption under certain circumstances, maintaining endogenous bone formation (Marie, 1996), augmenting vertebral bone mass without triggering mineralization defects (Grynpas et al., 1996), and enhancing osteoblast replication and bone formation *in vitro* (Omdahl and Deluca, 1973). Boivin et al. (1996), studied the biodistribution of Sr in monkeys following the administration of Sr ranelate via X-ray microanalysis. Changes in bone crystallinity were assessed using X-ray diffraction and Raman micro-spectroscopy. Sr enters the bone mineral of trabecular and cortical bones in a dose-dependent manner, predominantly into new bones, and mainly into trabecular bones. Upon drug withdrawal, the concentration of Sr in the bone rapidly decreases, distinguishing this drug, which is now developed as a medication, from bisphosphonates that remain permanently in the bone. No changes in lattice, crystallinity, or crystal structure were observed. In each crystal, less than one in ten Ca ions is replaced by a Sr ion. Long-term studies in rats confirmed significant increases in trabecular bone volume, mineralized bone volume, and osteoblast surfaces, with a decrease in osteoclast numbers, but bone thickness was not affected (Escanero and Córdova, 1991).

The synthetic activity of Sr in bone metabolism, especially in the context of osteoporosis treatment, has become a significant focus in recent research (Saidak and Marie, 2012). Studies have demonstrated that Sr influences bone turnover, marked by simultaneous increases in bone formation and bone resorption (Raisz, 2005; Seeman and Delmas, 2006; Mentaverri et al., 2012). Osteoporosis, predominantly affecting postmenopausal women, is characterized by low bone mass and heightened fragility, leading to an increased risk of fractures (Seeman and Delmas, 2006). This condition often results from estrogen deficiency, which disrupts the delicate balance between bone resorption and formation (Parfitt, 1984). Strontium ranelate, recognized for its efficacy in treating osteoporosis, is particularly recommended for severe cases in postmenopausal women and elderly men, serving as a second-choice medication. Its dual-action mechanism, which involves decreasing bone resorption while stimulating bone formation, enhances bone mineral density and reduces fracture risks (Fogelman and Blake, 2005; Deeks and Dhillon, 2010; Boivin et al., 2012). The standard dosage is 2 g daily, taken orally, and it is advised to be consumed apart from calcium-rich foods or supplements for better absorption. While undergoing strontium ranelate therapy, it's essential to ensure sufficient vitamin D and calcium intake for optimal bone health. The safety profile of strontium ranelate is generally favorable, but monitoring for adverse reactions such as nausea, diarrhea, headache, and dermatologic responses is vital (Fassbender et al., 2006). Regular assessments of bone mineral density are recommended to gauge the treatment’s effectiveness. Patients with a history of cardiovascular events should exercise caution when using strontium ranelate, as research indicates a significant proportion of osteoporosis patients on this medication have pre-existing cardiovascular risk factors (Yu et al., 2015). Therefore, individual patient factors, such as osteoporosis severity, comorbidities, and concurrent medications, are crucial considerations in strontium ranelate’s clinical application. In conclusion, strontium ranelate presents as a viable osteoporosis treatment, especially for postmenopausal women and elderly men, but its use requires careful evaluation and monitoring in consideration of the patient’s overall health and associated risks.

Furthermore, research has shown that strontium ranelate significantly reduces the incidence of hip and vertebral compression fractures in postmenopausal osteoporosis (Reginster et al., 2002; Meunier et al., 2004). The mechanism by which strontium ranelate treats osteoporosis involves enhancing endogenous osteoblast replication and bone formation, thus affecting bone remodeling (Canalis et al., 1996). Strontium ranelate acts as an osteoblast stimulant, activating the calcium-sensing receptor (CaSR), a G-protein-coupled receptor located in bone cells and associated with bone remodeling (Brown, 2003). While Sr ions have a lower affinity than Ca ions, they still function as agonists for the calcium-sensing receptor in bone. It has been confirmed that the calcium-sensing receptors in osteoblasts activated by Sr might exhibit different functionalities compared to other calcium-sensing receptors (Quarles, 1997).

### 4.4 Association between strontium and type 2 diabetes mellitus

Tremendous studies have demonstrated Sr treatment could alleviate oxidative damage (Yalin et al., 2012). There’s a negative correlation between the level of Sr in the blood and lipid peroxidation and oxidative stress (Barneo-Caragol et al., 2018a), with Sr offering protection at the chromosomal level (Bai et al., 2016). Sr may play a part in anti-oxidation and lipid metabolism (Yalin et al., 2012; Bai et al., 2016; Barneo-Caragol et al., 2018c), and it is associated with the development of T2DM (Maehira et al., 2011; SAidak et al., 2012; Vidal et al., 2013). Therefore, Sr could potentially be a promoting factor in the treatment of diabetes. However, there is still a lack of evidence to support this claim from animal studies to humans. Calcium, as a fundamental element, plays a critical role in biological functions and is associated with diabetes (Becerra-Tomás et al., 2014; Lorenzo et al., 2014; Rooney et al., 2016). Based on the similar chemical and biological properties of Sr and Ca, it has been suggested that they might have synergistic or competitive effects (Pors Nielsen, 2004). The ratio of Ca to Sr (Ca/Sr) could indicate to some extent the balance between Ca and Sr. In healthy populations, the Ca/Sr in bones is similar to that in serum, typically ranging from 1000:1 to 2000:1 (Wongdee et al., 2017). The balance between Sr and Ca mainly depends on intestinal absorption and bone turnover. Whether this balance would be disrupted under diabetic conditions remains unknown. Surveys have shown an inverse relationship between plasma Sr and T2DM and impaired glucose regulation (IGR). This association is independent of sociodemographic factors, lifestyle, disease conditions, and multiple plasma minerals. As plasma Sr increases, the odds of T2DM decrease significantly, then reach a plateau. The concentration of Sr in plasma is also inversely related to IGR, but this relationship is somewhat weakened.

Contrary to previous studies, the research results on the relationship between Sr and T2DM have been conflicting (Hansen et al., 2017; Li et al., 2017; Simić et al., 2017). A domestic study showed an increase in plasma Sr levels in patients with T2DM (Li et al., 2017). However, two Norwegian studies showed no difference in blood Sr levels between patients with T2DM and the control group (Hansen et al., 2017; Simić et al., 2017). This discrepancy may be related to the Sr concentration reported in the Norwegian studies, which was much lower (about 18–19 mg/L) compared to our results and other studies. The cause of this discrepancy could be due to population heterogeneity or dietary differences.

Additionally, skalnaya et al., 2018, and colleagues found an inverse relationship between serum Sr levels and insulin resistance in 80 women with pre-diabetes. Given the limited sample size of these studies, and that potential confounding factors were not fully considered in the analyses, these results should be interpreted with caution.

Over the past decade, extensive research has been conducted on the effects of Sr on bones. However, little is known about the biological mechanisms of Sr in the development of diabetes (Hellman et al., 1997; Maehira et al., 2011). *In vitro* studies show that Sr has potential anti-diabetic effects. Experimental evidence suggests that in an osteoporotic rat model (Yalin et al., 2012), treatment with Sr ranelate can lower the level of lipid peroxidation index (MDA), and increase the activity of superoxide dismutase, glutathione peroxidase, and catalase. Sr exhibits anti-diabetic effects by regulating the expression of relevant genes in the pancreas, reducing blood sugar levels, and improving tolerance to insulin, leptin, and adiponectin. Additionally, Sr has analgesic effects, involving reducing inflammatory cytokines (TNF-alpha and interleukin-1b) via an opiate-dependent mechanism (De Melo Nunes et al., 2015). While Sr has been shown to have anti-inflammatory effects through an opioid-like mechanism, it's unclear whether it plays a role in blood sugar control via opioid signaling pathways. Evidence from animal studies suggests that Sr might be involved in anti-adipogenesis, possibly by activating peroxisome proliferate-activated receptors in adipocytes. Lipid metabolism disorder, inflammation, and oxidative stress are key pathogenic mechanisms for insulin resistance and the development of diabetes (Savage et al., 2007; Zheng et al., 2018). Therefore, Sr’s potential antioxidant, anti-inflammatory, and anti-adipogenic effects may offer protective benefits against diabetes.

The epidemiological evidence of Sr’s impact on lipid parameters and oxidative stress is limited and mainly comes from small sample-size studies (Barneo-Caragol et al., 2018b). We evaluated the relationship between plasma Sr and lipid peroxidation and lipid parameters in NGT (Normal Glucose Tolerance), IGR (Impaired Glucose Regulation), and T2DM. An inverse relationship was observed between plasma Sr and total cholesterol, low-density lipoprotein cholesterol (LDL-C), and lipid peroxidation index (MDA). However, contrary to previous studies, there was no significant correlation between Sr and total cholesterol, low-density lipoprotein cholesterol (LDL-C), and the lipid peroxidation index (MDA). Also, the relationship between Sr and fasting blood glucose, fasting insulin, or HOMA (Homeostatic Model Assessment) was not significant. This suggests that while Sr could potentially have impacts on cholesterol levels and lipid peroxidation, its relationship with these markers and blood sugar regulation might be complex and is not fully understood at this time. Further research with larger sample sizes and more diverse populations would be needed to clarify these relationships and to understand the potential role of Sr in metabolic health and disease. Sr and Ca have long been considered to behave similarly and to have a synergistic effect due to their similar chemical properties. However, Sr has been shown to interact with Ca channels and to regulate intracellular Ca levels through calcium-sensing receptors (Fernández et al., 2013). Previous research has shown a positive correlation between serum calcium levels and the risk of T2DM, with the relationship between serum calcium and T2DM risk dependent on estimates of either total serum calcium or albumin-corrected serum calcium. This suggests that alterations in calcium homeostasis might be involved in the development of T2DM. The interaction between Sr and Ca, therefore, could potentially have an impact on T2DM risk as well, although further research is needed to fully understand these relationships. The exact mechanism of how Sr influences Ca metabolism and how this might relate to diabetes risk is still not fully clear (Becerra-Tomás et al., 2014; Lorenzo et al., 2014; Rooney et al., 2016).

### 4.5 Association between strontium and hypertension

Pregnancy-induced hypertension (PIH), including gestational hypertension (GH) and preeclampsia (PE), is considered one of the main causes of morbidity and mortality in pregnant and perinatal women (Helewa et al., 1997; Savitz et al., 2014; Mol et al., 2016). It has detrimental effects on the future lives of these mothers and children (Ananth and Basso, 2010; Bakker et al., 2011; Veerbeek et al., 2015; Alsnes et al., 2017). Pregnant women with PIH are at a higher risk of complications such as cardiovascular diseases (Veerbeek et al., 2015; Leon et al., 2019), while infants born to women with PIH may be at risk of adverse birth outcomes such as preterm birth (Clausson et al., 1998; Bakker et al., 2011). Increasing evidence supports the idea that oxidative stress, especially placental oxidative stress, is a major factor leading to PIH (Hilali et al., 2013; Kintiraki et al., 2015; Lin et al., 2016; Mol et al., 2016). A study conducted by Tang and colleagues (Tang et al., 2021) surveyed 5432 pregnant women and found a significant correlation between increased risk of gestational hypertension and lower levels of urinary Sr. The correlation between strontium (Sr) levels and the prevention of gestational hypertension appears to be particularly significant in pregnant women under the age of 35. This suggests that Sr may be significantly effective in reducing the likelihood of high blood pressure development. Trace elements are vital components of the body’s antioxidant defense system (Mbofung et al., 1986; Evans and Halliwell, 2001). Sr, one of these trace elements, exhibits particular biological effects in the human antioxidant defense system. It naturally exists in the environment and is effectively absorbed into the human body through the soil-plant-food chain. The elimination of Sr largely depends on age (Pors Nielsen, 2004). Sr is primarily excreted from the kidneys, and its presence in urine can reflect internal exposure levels (Dahl et al., 2001). Previous research points out that Sr can reduce oxidative stress by elevating the levels of superoxide dismutase (SOD) and catalase (CAT), which are two enzymes that clear free oxygen radicals in the body (El-Megharbel et al., 2015).

Studies suggest that Sr is linked to the pathophysiology of Pregnancy-induced hypertension (PIH) (Barneo-Caragol et al., 2018a; Barneo-Caragol et al., 2018b). An observational study revealed elevated serum Sr concentrations and oxidative status in a small portion of preeclampsia (PE) patients. Researchers such as Barneo-Caragol argue that the increase in serum Sr levels may restore the balance between bodily defense and oxidative damage, suggesting a potentially beneficial role for Sr in oxidative damage. Moreover, studies indicate that maternal age is associated with the metabolism of Sr (Dietl and Farthmann, 2015; Shagina et al., 2015). However, there is currently insufficient evidence to support the relationship between Sr levels and PIH under varying maternal ages.

### 4.6 Association between strontium and cardiovascular disease

The role of Sr in cardiovascular health has been illustrated by numerous studies (Corbett et al., 2014). Dawson et al. (1978), showed that Sr is related to cardiovascular construction and function, Sr reduces mortality from cardiovascular diseases, abnormal sodium/Sr ratio is associated with a variety of cardiovascular diseases, drinking water sodium/Sr ratio and urinary sodium/Sr ratio are significantly positively and negatively correlated with atherosclerosis, and the mechanism of action may be that Sr is competitively absorbed with sodium in the intestine, and the presence of Sr reduces the absorption of sodium in the body and increases sodium excretion in the body. Tang et al., 2021, studied 5432 pregnant women and found that increased risk of gestational hypertension was significantly associated with lower urinary Sr levels, and this correlation was more pronounced in younger pregnant women under 35 years of age, suggesting that Sr plays an important role in preventing the development of gestational hypertension.

## 5 Health effects of strontium Overdose and deficiency

Sr has a lower toxicity than Ca. However, in experimental animals, high levels of Sr in the diet can induce skeletal changes akin to rachitic lesions, especially in conditions of low Ca intake (Bartley and Reber, 1961; COLVIN et al., 1972). This phenomenon is caused by a combination of impaired intestinal absorption of Ca and a decrease in 1,25-dihydroxycholecalciferol produced by the kidneys. The direct impact of Sr on intestinal Ca absorption may be due to shared absorption pathways between these two metals, with both active and passive transport mechanisms favoring Ca absorption. This competitive inhibition has been confirmed in isolated intestinal slices and perfused intestines. It has been shown that calcium-binding proteins bind Sr to a lesser extent than Ca (Schrooten et al., 1998), and Sr inhibits renal 1-hydroxylase, impairing the production of 1,25-(OH)_2_ Vitamin D_3_ (Omdahl and Deluca, 1973). Reports indicate that in chickens, Vitamin D_3_ and its 24-hydroxy derivative lost their anti-rachitic activity under high dietary Sr concentrations, but 1,25-dihydroxy vitamin D retained its ability to induce the synthesis of calcium-binding proteins in intestinal epithelial cells and stimulated bone mineralization to some extent (Bauman et al., 1988). High-calcium and high-Sr diets in pregnant mice reduced the content of calcium-binding proteins in maternal intestines and placenta, which could eventually lead to the hypomineralization of the fetal skeleton (Bruns et al., 1982). Animal studies suggest that young animals are more sensitive to excessive Sr than older animals, and inadequate intake of Ca and Vitamin D can increase the detrimental effects on the skeleton.

Ozgür et al. (1996), published an observational study on the importance of Sr for human nutrition. They found that in two regions of Turkey where the soil content of Sr greatly varied, there was a high prevalence of rickets. In Region 1, where Sr content was above 350 ppm, the prevalence of rickets was 31.5%; in Region 2, where Sr content was below 350 ppm, the prevalence of rickets was 19.5%. Therefore, high concentrations of Sr in the soil may induce rickets, although lack of sunlight and dietary deficiencies may also be contributing factors. Verberckmoes et al. (2003), demonstrated the direct impact of high-dose Sr on bone *in vitro*, finding evidence of insufficient formation of hydroxyapatite under high and medium doses of Sr, which may resemble the situation of osteoporosis in dialysis patients. Although the prevalence of osteoporosis has decreased in recent years, there are indeed less than 5% of dialysis patients suffering from osteoporosis. It is well known that the concentration of Sr in bone is elevated, and the Sr/Ca ratio in bone is high, which may be due to the high concentration of Sr in dialysis fluid (Cohen-Solal et al., 2002). However, patients with end-stage renal failure who have not yet undergone dialysis have normal bone Sr levels, but the prevalence of osteoporosis is still high (Spasovski et al., 2003).

It has been found that the ingestion of toxic doses of Sr leads to defective bone mineralization, similar to rickets/osteoma Acia, and the effect is more pronounced in animals consuming a low-calcium diet (Omdahl and Deluca, 1971; Morohashi et al., 1994). This may be related to Sr’s impact on parathyroid hormone and vitamin D_3_ levels, as well as Sr’s direct integration into bone.

## 6 Conclusion and future directions

Current research on Sr effect in human health, while enlightening, reveals a landscape replete with unanswered questions and unexplored avenues. The existing body of work, primarily observational and experimental, underscores the potential of Sr in mitigating various non-communicable diseases, yet it falls short of establishing definitive causal relationships. Notably, the intricacies of Sr metabolism, its optimal dietary levels, and long-term health implications remain inadequately understood.

The road ahead demands a concerted effort to expand robust epidemiological data and conduct controlled clinical trials. There is a critical need to quantify the dietary intake of Sr accurately and establish reference values, considering the variability across different populations and dietary habits. Future studies should aim to unravel the mechanistic pathways of Sr at the molecular level, providing insights into its therapeutic potential and safety profile. Furthermore, understanding the interaction of Sr with other trace elements and nutrients, and how these relationships influence overall health, will be vital. Such comprehensive research efforts will not only illuminate the nuanced role of Sr in human health but also guide dietary recommendations and potential therapeutic applications, paving the way for preventive and treatment strategies for chronic diseases.

This review culminates in a nuanced understanding of strontium’s multifaceted role in human health. The evidence presented herein highlights the significant potential of Sr in influencing various health outcomes, particularly in the context of chronic diseases. However, the journey to fully comprehend and harness Sr’s benefits is far from complete. Such endeavors will not only solidify our understanding of the role of health and disease but also inform public health policies and clinical practices. In conclusion, while the current evidence is promising, it serves as a springboard for future research, crucial for unlocking the full potential of Sr in enhancing human health.
